# Supplementary material for: Factors associated with low back pain among construction workers in Nepal: A cross-sectional study
Source: PLoS One. 2021 Jun 1;16(6):e0252564. doi: 10.1371/journal.pone.0252564 (PMC8168885; doi:10.1371/journal.pone.0252564)
Supplement: Attachment — Submitted filename: Plos article.pdf [file pone.0252564.s001.pdf]

PLOS ONE

Factors associated with low back pain among construction workers in Nepal: A cross-sectional study

--Manuscript Draft--

**Manuscript Number:**	PONE-D-21-04117
**Article Type:**	Research Article
**Full Title:**	Factors associated with low back pain among construction workers in Nepal: A cross-sectional study
**Short Title:**	Factors associated with low back pain among construction workers in Nepal

**Corresponding Author:**	Bikram Adhikari<br>BP Koirala Institute of Health Sciences<br>Panauti, Bagmati NEPAL

**Keywords:**	Low back pain; Construction workers; Nepal; Factors

**Abstract:**	Background: Low Back Pain (LBP) is the commonest cause of disability throughout the world. This study aimed to determine the prevalence of LBP and factors associated with it among construction workers in Nepal.<br>Methods: A community-based cross-sectional study was conducted among construction workers working in purposively selected, Banepa and Panauti municipalities of Kavre district, from September 2019 to February 2020. Data was collected by face-to-face interview from 402 eligible participants from all wards of both municipalities using semi-structured questionnaire. Mobile-based data collection was done using KoboCollect. Data were exported to and analysed using R-programming software (R-3.6.2). Univariate and multivariate logistic regressions were performed at 95% confidence interval (CI).<br>Result: The one-year prevalence of LBP and CLBP among construction workers were 52.0% (95%CI: 47.0-57.0) and 14.2% (95%CI: 10.9-18.0) respectively. Higher odds of having LBP was found among females [Adjusted Odds Ratio (AOR) =2.42; 95%CI: 1.12-5.23], those living below poverty-line (AOR=2.35; 95%CI: 1.32-4.19), those with more than five years of work experience (AOR=1.66; 95%CI: 1.01-2.73) and those with intermediate sleep quality (AOR=2.06; CI: 1.03-4.11). About 80.0% of construction workers with LBP did not seek healthcare services because of time constraints (90.9%), financial constraints (18.1%) and fear of opportunity cost (40.9%). The majority of the participants (94.8% among those without LBP and 72.3% among those with LBP) did nothing to prevent or manage LBP.<br>Conclusion: The prevalence of LBP in the past one year was high among construction workers and a high proportion of workers did nothing to prevent or manage LBP. Public health professionals should set up health promotion, education, and intervention aimed at increasing awareness of preventive techniques and predisposing factors of LBP.

**Order of Authors:**	Bikram Adhikari
	Nilambar Jha
	Rajendra Karkee
	Archana Shrestha
	Roshan Dhakal
	Aarju Niraula
	Sangita Majhi
	Antesh Kumar Pandit
	Niroj Bhandari
	Anup Ghimire

**Additional Information:**	
**Question**	**Response**

Powered by Editorial Manager® and ProduXion Manager® from Aries Systems Corporation

**Financial Disclosure**<br><br>Enter a financial disclosure statement that describes the sources of funding for the work included in this submission. Review the submission guidelines for detailed requirements. View published research articles from *PLOS ONE* for specific examples.<br><br>This statement is required for submission and **will appear in the published article** if the submission is accepted. Please make sure it is accurate.<br><br>**Unfunded studies**<br>Enter: *The author(s) received no specific funding for this work.*<br><br>**Funded studies**<br>Enter a statement with the following details:<br>• Initials of the authors who received each award<br>• Grant numbers awarded to each author<br>• The full name of each funder<br>• URL of each funder website<br>• Did the sponsors or funders play any role in the study design, data collection and analysis, decision to publish, or preparation of the manuscript?<br>• **NO** - Include this sentence at the end of your statement: *The funders had no role in study design, data collection and analysis, decision to publish, or preparation of the manuscript.*<br>• **YES** - Specify the role(s) played.<br><br>* typeset	The author(s) received no specific funding for this work.
**Competing Interests**<br><br>Use the instructions below to enter a competing interest statement for this submission. On behalf of all authors, disclose any competing interests that could be perceived to bias this work—acknowledging all financial support and any other relevant financial or non-financial competing interests.<br><br>This statement **will appear in the published article** if the submission is accepted. Please make sure it is	The authors have declared that no competing interests exist.

accurate. View published research articles from *PLOS ONE* for specific examples.<br><br>**NO authors have competing interests**<br><br>Enter: *The authors have declared that no competing interests exist.*<br><br>**Authors with competing interests**<br><br>Enter competing interest details beginning with this statement:<br><br>*I have read the journal's policy and the authors of this manuscript have the following competing interests: [insert competing interests here]*<br><br>* typeset	
**Ethics Statement**<br><br>Enter an ethics statement for this submission. This statement is required if the study involved:<br><br>• Human participants<br>• Human specimens or tissue<br>• Vertebrate animals or cephalopods<br>• Vertebrate embryos or tissues<br>• Field research<br><br>Write "N/A" if the submission does not require an ethics statement.<br><br>General guidance is provided below. Consult the submission guidelines for detailed instructions. **Make sure that all information entered here is included in the Methods section of the manuscript.**	Institutional Review Committee (IRC) of B.P Koirala Institute of health Sciences, Dharan, Nepal provided ethical approval.<br>The IRC approval number of this study is: 057/076/077-IRC.Written informed consent was obtained from all the participants of the study.

**Format for specific study types**<br><br>**Human Subject Research (involving human participants and/or tissue)**<br>• Give the name of the institutional review board or ethics committee that approved the study<br>• Include the approval number and/or a statement indicating approval of this research<br>• Indicate the form of consent obtained (written/oral) or the reason that consent was not obtained (e.g. the data were analyzed anonymously)<br><br>**Animal Research (involving vertebrate animals, embryos or tissues)**<br>• Provide the name of the Institutional Animal Care and Use Committee (IACUC) or other relevant ethics board that reviewed the study protocol, and indicate whether they approved this research or granted a formal waiver of ethical approval<br>• Include an approval number if one was obtained<br>• If the study involved *non-human primates*, add *additional details* about animal welfare and steps taken to ameliorate suffering<br>• If anesthesia, euthanasia, or any kind of animal sacrifice is part of the study, include briefly which substances and/or methods were applied<br><br>**Field Research**<br><br>Include the following details if this study involves the collection of plant, animal, or other materials from a natural setting:<br>• Field permit number<br>• Name of the institution or relevant body that granted permission	
**Data Availability**<br><br>Authors are required to make all data underlying the findings described fully available, without restriction, and from the time of publication. PLOS allows rare exceptions to address legal and ethical concerns. See the PLOS Data Policy and FAQ for detailed information.	Yes - all data are fully available without restriction

Powered by Editorial Manager® and ProduXion Manager® from Aries Systems Corporation

A Data Availability Statement describing where the data can be found is required at submission. Your answers to this question constitute the Data Availability Statement and **will be published in the article**, if accepted.<br><br>**Important:** Stating 'data available on request from the author' is not sufficient. If your data are only available upon request, select 'No' for the first question and explain your exceptional situation in the text box.<br><br>Do the authors confirm that all data underlying the findings described in their manuscript are fully available without restriction?	
**Describe where the data may be found in full sentences. If you are copying our sample text, replace any instances of XXX with the appropriate details.**<br><br>• If the data are **held or will be held in a public repository**, include URLs, accession numbers or DOIs. If this information will only be available after acceptance, indicate this by ticking the box below. For example: *All XXX files are available from the XXX database (accession number(s) XXX, XXX.)*.<br>• If the data are all contained **within the manuscript and/or Supporting Information files**, enter the following: *All relevant data are within the manuscript and its Supporting Information files.*<br>• If neither of these applies but you are able to provide **details of access elsewhere**, with or without limitations, please do so. For example:<br><br>*Data cannot be shared publicly because of [XXX]. Data are available from the XXX Institutional Data Access / Ethics Committee (contact via XXX) for researchers who meet the criteria for access to confidential data.*<br><br>*The data underlying the results presented in the study are available from (include the name of the third party*	All relevant data are within the manuscript and its Supporting Information files.

Powered by Editorial Manager® and ProduXion Manager® from Aries Systems Corporation

and contact information or URL).<br>• This text is appropriate if the data are owned by a third party and authors do not have permission to share the data.<br><br>* typeset	
Additional data availability information:	Tick here if the URLs/accession numbers/DOIs will be available only after acceptance of the manuscript for publication so that we can ensure their inclusion before publication.

Manuscript
Click here to access/download;Manuscript;Original manuscript_BA.docx

Factors associated with low back pain among construction workers in Nepal: A cross-sectional study

Bikram Adhikari [1*], Nilambar Jha [1], Rajendra Karkee [1], Archana Shrestha [2,3,4], Roshan Dhakal [1], Aarju Niraula [1], Sangita Majhi [1], Antesh Kumar Pandit[1], Niroj Bhandari [2,4], Anup Ghimire [1]

[1] School of Public Health and Community Medicine, B.P. Koirala Institute of Health Sciences (BPKIHS), Dharan,Sunsari, Nepal

[2] Department of Public Health, Kathmandu University School of Medical Sciences, Dhulikhel, Nepal

[3] Department of Chronic Disease Epidemiology, Yale School of Public Health, New Haven, USA

[4] Institute of Implementation Science and Health

[*] Corresponding Author

Email: bikram.adhikariadhitya@gmail.com

Abstract

Background: Low Back Pain (LBP) is the commonest cause of disability throughout the world. This study aimed to determine the prevalence of LBP and factors associated with it among construction workers in Nepal.

Methods: A community-based cross-sectional study was conducted among construction workers working in purposively selected, Banepa and Panauti municipalities of Kavre district, from September 2019 to February 2020. Data was collected by face-to-face interview from 402 eligible participants from all wards of both municipalities using semi-structured questionnaire. Mobile-based data collection was done using KoboCollect. Data were exported to and analysed using R-programming software (R-3.6.2). Univariate and multivariate logistic regressions were performed at 95% confidence interval (CI).

Result: The one-year prevalence of LBP and CLBP among construction workers were 52.0% (95%CI: 47.0-57.0) and 14.2% (95%CI: 10.9-18.0) respectively. Higher odds of having LBP was found among females [Adjusted Odds Ratio (AOR) =2.42; 95%CI: 1.12-5.23], those living below poverty-line (AOR=2.35; 95%CI: 1.32-4.19), those with more than five years of work experience (AOR=1.66; 95%CI: 1.01-2.73) and those with intermediate sleep quality (AOR=2.06; CI: 1.03-4.11). About 80.0% of construction workers with LBP did not seek healthcare services because of time constraints (90.9%), financial constraints (18.1%) and fear of opportunity cost (40.9%). The majority of the participants (94.8% among those without LBP and 72.3% among those with LBP) did nothing to prevent or manage LBP.

Conclusion: The prevalence of LBP in the past one year was high among construction workers and a high proportion of workers did nothing to prevent or manage LBP. Public health

professionals should set up health promotion, education, and intervention aimed at increasing awareness of preventive techniques and predisposing factors of LBP.

Key Words: *Low back pain; Construction workers; Nepal; Factors*

Introduction

Nepal is an agrarian society where most people are dependent on works requiring physical capabilities like farming. Majority of the population of Nepal have informal employment dependent on traditional agricultural practices, even within the formally employed population, agriculture and construction sectors are the major areas of employment[1]. According to the national labor survey, approximately 2.2 million workers in Nepal are engaged in the construction sector which is estimated to constitute around 10-11 percent of the Gross Domestic Product (GDP) in Nepal [21]. Socio-economically poor people from rural areas are engaged in this sector[22].

Construction work is renowned as unhealthy because of the high mechanical nature and hard physical labor involved [18,19]. A construction manual worker is a general/blue-collar worker employed in the construction industry and works predominantly on construction sites and is typically engaged in hands-on aspects of the industry other than design or finance. This includes members of specialist trades such as builders, electricians, carpenters, bricklayer, manual labor, armature fixing workers, internal finish workers and plumbers [20]

Construction workers are at a higher risk of musculoskeletal conditions [10] with low back pain, a common problem among construction workers [11,12] as nearly 80% working postures were found harmful for the musculoskeletal system of the construction workers [13]. Low back pain was found in about half of the respondents in various studies done among construction workers [14–16]. Many construction manual workers may suffer from low back pain but do not report it as an injury [17].

Low back pain (LBP) is the most prevalent musculoskeletal condition and a leading cause of disability throughout the world [2]. It is one of the least prioritized non communicable diseases in Nepal [3]. Low back pain (LBP) causes disability, severe pain and extended sick leave affecting about 80 % of individuals during their lifetime[4,5]. LBP leads to high direct and indirect costs which have great medical, social and economic impacts for individuals, families, society, and government [6–9].

A large proportion of Nepalese is engaged in the construction sector which has a bad reputation due to the high mechanical nature and hard physical labor involved. The health of the construction workers is an important issue that needs to be addressed for the development of the country. Little is known about low back pain and factors associated with low back pain among construction workers of Nepal. In addition, there are very few studies to understand about measures taken by construction workers to prevent and manage low back pain.

Here, we aimed to estimate the prevalence of low back pain among construction workers and associated risk factors and measures taken by construction workers to prevent and manage low back pain.

Methods

Study Area and Study Participants

We conducted a cross sectional study between September, 2019 and February, 2020 in Kavre district of central Nepal. Two out of 13 municipalities in Kavre district, Panauti and Banepa, were purposively selected. Principal investigator visited every ward of each municipalities and located building construction sites with the assistance of local people. All people working in construction sites which researcher located within study duration and who meet eligibility

criteria were enrolled in the study. The inclusion criteria to enrol participants were: a) building construction workers aged 18 years or above and b) work experience of one year or more. The exclusion criteria were: a) building construction workers who were not able to communicate clearly and b) those who did not give consent.

Data collection:

Data were collected through face-to-face interviews and anthropometric measurements.

Interview: Data collection was done using a semi-structured questionnaire which included the following components:

- A questionnaire assessing socio-demographic characteristics of participants like age, gender, religion, ethnicity, marital status, and average monthly family income; lifestyle characteristics and occupational characteristics.
- Extended Nordic Musculoskeletal Questionnaire to assess the low back pain. Extended Nordic Musculoskeletal Questionnaire (NMQ-E) was a structured and validated tool that had been used widely across various study settings. The validity of the questionnaire was done by three experts: an epidemiologist, orthopedic surgeon, and community medicine expert.
- Questions addressing psychosocial factors like job insecurity, work-family balance, job satisfaction and exposure to the hostile work environment. DASS-21 was used to assess the depression, anxiety, and stress.

Anthropometric measurement: Height and weight were measured using the digital weighing machines and the portable measuring tape, respectively, from which body mass index (BMI) was calculated. The BMI was calculated using the formula: weight (kg) / height (cm) ^{2}.

Operational Definitions:

Low back pain: Low back pain (LBP) is said to be present if the participant report pain and discomfort localized below the costal margin and above the inferior gluteal folds, with or without leg pain [8].

Chronic Low Back Pain: Chronic back pain is the low back pain persisting for more than 12 weeks or after the period of healing or recurring back pain that intermittently affects an individual over a long period of time[8]. In the present study, Chronic Low Back pain (CLBP) was assessed through a question item asking if a person experienced low back pain in the past three months prior to the interview.

Poverty: Those participants whose family members living with less than $1.90 per day were considered to be living below the poverty line[23].

Obesity: Participants were classified into Underweight, Normal, Overweight and Obese based on Asia-Pacific guidelines for obesity classification[24].

Current Smoker: Current smokers were defined as those who reported smoking any tobacco product within the last 30 days. Respondents who reported smoking at least 100 cigarettes in their lifetime and who, at the time of the survey, did not smoke were defined as past smokers [25].

Current Alcohol Drinkers: Those who consumed alcohol within the last 30 days were considered current alcohol drinkers [25].

Sleep duration: Sleep duration was assessed by asking two questions-“What time you usually go to sleep at night? And what time you usually wake up in the morning?” Later based on the response, sleep duration was coded into four categories: (a) 3-4 hours, (b) 5-6 hours, (c) 7-8 hours, and (d) 9 hours and more (24).

Sleep quality: It was measured by two questions—1) “In the past one year, how do you rate your own sleep quality?” The response were a) Good b) Intermediate c) Poor and 2) Do you think you sleep enough? The response was either yes or no [26].

Co-morbidities: The participants were asked whether they had any kind of existing diseases like diabetes, hypertension, stones or others. Those who had existing diseases were considered for the presence of co-morbid conditions.

Type of construction work: Based on several pieces of literature, the construction workers were classified into manual labors, bricklayer, plumber, electrician, painter, carpenter, interior finish worker, scaffolders, armature fixing worker and others.

Employment status: It is to determine whether the employment status is seasonal or permanent.

Work experience: Duration of work experience was assessed by asking two questions—1) At what age have you started working in the construction industry? and 2) How long have you been doing construction work?

Depression, Anxiety and Stress: Depression, anxiety and stress were assessed using Depression, Anxiety and Stress Scale (DASS-21) developed by Lovibond *et al* [27]. The internal consistency of the DASS-21 Nepali version was 0.77 for DASS-depression; 0.80 for DASS-anxiety; and 0.82 for DASS-stress, which indicates good Cronbach's alpha values [28].

Job satisfaction: It was measured by the questions- "Please tell me whether you: strongly agree, agree, disagree, or strongly disagree with this statement: I am satisfied with my job". Responses of "strongly disagree" and "disagree" were defined as low job satisfaction [29].

Work-family imbalance: It was measured by the following question: "Please tell me whether you: strongly agree, agree, neutral, disagree, or strongly disagree with this statement: It is easy for me to combine work with family responsibilities." Responses of "strongly disagree" and "disagree" were defined as high work-family imbalance[29].

Exposure to the hostile work environment: It was measured by the question "During the past 12 months were you threatened, bullied, or harassed by anyone while you were on the job?" The response of "Yes" was defined as exposure to a hostile work environment[29].

Job insecurity: Job insecurity was measured by the question: "Please tell me whether you: strongly agree, agree, disagree, or strongly disagree with this statement: I am worried about becoming unemployed." Responses of "strongly agree" and "agree" were defined as high job insecurity [29].

Data Collection

The questionnaire form was created in the Kobotoolbox platform (form can be visualized in the internet link: https://ee.kobotoolbox.org /x/#Kz2aVRXw). Principal Investigator collected

data with android mobile phone using KoboCollect software and synchronized collected data into the cloud of Kobotoolbox.

Data collected in the mobile phone was monitored for its completeness after individual data entry. Proper validation and checks of the questionnaire were setup in KoboCollect to provide assurance of uniform and correct data entry. Ten percent of the collected data were cross-checked for cleanliness, errors, and problems at the end of every week.

The data synchronized into Kobotoolbox account was exported to R-programming software (R-3.6.2) for cleaning, coding, categorizing and to check completeness, consistence and outliers.

Statistical Analysis

Data was analysed using R-programming software (R-3.6.2) after completing pre-analysis tasks. Descriptive analysis of socio-demographic and occupational characteristics and factors related to LBP was done. Means and standard deviations were displayed for normally distributed numerical variables, while medians and inter quartile ranges (IQR) were displayed for non-normally distributed numerical variables. Frequency and percentages were displayed for categorical variables. The confidence interval around the prevalence was determined using the Clopper-Pearson method. Student t test or Mann Whitney U test were applied to compare numerical data between two groups. Bivariate and multivariate logistic regression analysis were carried out to determine the association of factors with low back pain. Throughout the

study, all tests were two-tailed and carried out at 95% CI. P-value less than 0.05 was considered statistically significant.

Ethical Approval

Ethical approval was obtained from the Institutional Review Committee, BPKIHS (Reference Number: 057/076/077-IRC) before conducting the study. Oral consent and permission were obtained from the contractor to assess construction workers under his/her control and informed written consent were obtained from eligible construction workers before enrolling them into the study. Confidentiality and anonymity of the participants were maintained and assured throughout the study.

Results

Overall, 456 construction workers were assessed, of which eligible participants were 415. Among 456 participants, 9.0% were not eligible for the study because of age limitation (5.7%) and the language barrier (3.3%). Out of 415 participants, 13 (3.1%) declined to participate in the study. The response rate of the study was 96.9%.

Socio-demographic and Occupational Characteristics

A total of 402 construction workers were included in the study: the socio-demographic and occupational characteristics of the participants are shown in the Table 1. Of the total participants, 83.8% were males. The age of the participants ranged from 18 to 64 years with the mean age of 31.78±9.49 years. Most of the participants were Hindus (77.2%) followed by Buddhists (18.2). About three-fourth (76.9%) of the participants were married and 16% were illiterate. About one-fifth of the construction workers of the present study were living with less than $1.90 per day (Table 1).

Most of the participants were manual labor representing 41.3% followed by Bricklayer accounting for 25.9%. The working hours per week of the participants ranged from 15 to 90 hours per week with an average of 58.35 ± 19.44 hours per week. The resting hours per day ranged from 0.5 to 3 hours with a mean of 1.29 ± 0.44 hours per day. The experience of the workers ranged from 1 to 40 years with a median of 5 years (Table 1).

Table 1. Socio-demographic and occupational characteristics of construction workers (N=402)

Characteristics	n (%) or Mean±SD or Median(IQR)
**Gender**	
Male	337 (83.8)
Female	65 (16.2)
**Age (in years)**	31.78±9.49
**Nationality**	
Nepali	399 (99.3)
Indian	3 (0.7)
**Ethnicity**	
Chhetri	65 (16.2)
Brahmin	55 (13.7)
Newar	51 (12.7
Tamang, Sherpa, and Bhote	70 (17.4)
Magar	50 (12.4)
Rai and Limbu	19 (4.7)
Kami/Damai/Badi/Gaine	20 (5.0)
Madhesi Brahmin and Janajati	7 (1.7)
Tharu	49 (12.2)
Other	16 (4.0)
**Religion**	
Hindu	311 (77.4)
Buddhist	73 (18.2)
Islam	2 (0.5)
Kirat	12 (3.0)
Christian	4 (1.0)
**Education**	
No education	64 (15.9)
Some primary	104 (25.9)
Completed primary	45 (11.2)
Some secondary	74 (18.4)
Completed secondary	66 (16.4)

Above secondary	49 (12.2)
**Marital Status**	
Married	309 (76.9)
Unmarried	87(21.6)
Widowed	4 (1.0)
Separated	2 (0.5)
**Family Size**	5 (4, 6)
**Dependent family members**	3 (2, 4)
**Monthly income (participant)**	N.Rs.25000 (20000, 25250)
**Monthly family income**	N.Rs. 45000 (30000, 55000)
**Poverty**	
Below the poverty line	80 (19.9)
Above the poverty line	322 (80.1)
**Type of Construction work**	
Manual labor	166 (41.3)
Bricklayers	104 (25.9)
Internal finish worker	59 (14.7)
Armature fixing	49 (12.2)
Painter	14 (3.5)
Electrician	10 (2.5)
**Work Per Week (hours)**	58.35 ± 19.44
**Rest Per Day (hours)**	1.29 ± 0.44
**Age at joining construction industry (year)**	23.29±7.10
Less than 18 year	127 (31.6)
More than 18 year	275 (68.4)
**Work experience in the construction sector (years)**	5 (3, 11)
One year or less	51 (12.7)
2-10 years	246 (61.2)
11-20 years	81 (20.1)
Above 20 years	24 (6.0)

***N**: Total frequency; **n**: frequency; **%**: Percentage; **SD**: Standard Deviation;*

***IQR**: Interquartile Range*

Prevalence of Low Back Pain and Chronic Low Back Pain

Table 2 shows the overall and gender-wise distribution of low back pain The one-year prevalence of low back pain among construction workers was 52.0% (95% CI: 47.0-57.0) accounting highest prevalence among female, 72.3% (95%CI: 59.8-82.7) in comparison to males, 48.1% (95% CI: 42.6-53.6). The one-month, one-week and point prevalence were all higher among females in compared to males.

Table 2. Prevalence of low back pain among construction workers (N=402)

**Low back pain prevalence**	**Prevalence n[% (95% CI)]**		
	Overall(N=402)	Male(N=337)	Female(N=65)
One-year	209 [52.0 (47.0-57.0)]	162 [48.1 (42.6-53.6)]	47 [72.3 (59.8-82.7)]
One-month	130 [32.3 (27.8-37.2)]	98 [29.1 (24.3-34.2)]	32 [49.2 (36.6-61.9)]
One-week	115 [28.6 (24.2-33.3)]	85 [25.2 (20.7-30.2)]	30 [46.2 (33.7-59.0)]
Point prevalence	92 [22.9 (18.9-27.3)]	65 [19.3 (15.2-23.9)]	27 [41.5 (29.4-54.4)]

The prevalence of Chronic Low Back Pain was 14.2% (95% CI: 10.9-18.0) among construction workers accounting 11.9% (95% CI: 8.6-15.8) and 26.2% (95% CI: 16.0-38.5) among male and female respectively.

Factors associated with Low Back Pain

Univariate logistic regression analysis revealed significant factors associated with LBP were gender, age, marital status, poverty, perceived enough sleep, sleep quality, presence of comorbidity, work experience, anxiety and job insecurity (Table 3).

232 **Table 3. Univariate logistic regression to determine association of socio-demographic,**

233 **lifestyle, occupational and psychosocial factors with LBP (N=402)**

Variables	Low back pain, n (%)		Crude Odd's ratio (95% CI)	p-value
	Present	Absent		
**Socio-demographic factors**				
**Gender**				
Male (ref)	162 (48.1)	175 (51.9)	1	
Female	47 (72.3)	18 (27.7)	**2.82 (1.57-5.06)**	<0.001
**Age**				
18-30 years (ref)	90 (42.5)	122 (57.5)	1	
30-40 years	75 (60.5)	49 (39.5)	**2.08 (1.32-3.26)**	0.002
Above 40 years	44 (66.7)	22 (33.3)	**2.71 (1.52-4.84)**	0.001
**Marital status**				
Unmarried (ref)	24 (27.6)	63 (72.4)	1	
Married	182 (58.9)	127 (41.1)	**3.76 (2.23-6.34)**	<0.001
**Religion**				
Hindu (ref)	169 (54.3)	142 (45.7)	1	
Buddhism	34 (46.6)	39 (53.4)	0.72 (0.44-1.22)	0.233
Other	6 (33.3)	12 (66.7)	0.42 (0.15-1.15)	0.091
**Poverty**				
Below poverty line	53 (66.3)	27 (33.8)	**2.09 (1.25-3.49)**	0.004
Above poverty line (ref)	156 (48.4)	166 (51.6)	1	
**Lifestyle factors and comorbidities**				
**Comorbidities**				
Absent(ref)	167 (48.3)	179 (51.7)	1	
Present	42 (75.0)	14 (25.0)	**3.22 (1.70-6.10)**	<0.001
**Hypertension**				
Absent (ref)	145 (51.2)	138 (48.8)	1	
Present	64 (53.8)	55 (46.2)	1.11 (0.72-1.70)	0.641
**Obesity**				
Normal (ref)	100 (50.0)	100 (50.0)	1	
Underweight	15 (42.9)	20 (57.1)	0.75 (0.36-1.55)	0.436
Overweight	32 (50.8)	31 (49.2)	1.03 (0.59-1.82)	1.000
Obese	62 (59.6)	42 (40.4)	1.48 (0.91-2.38)	0.097
**Current smoking**				
No (ref)	112 (51.6)	105 (48.4)	1	

Yes	97 (52.4)	88 (47.6)	1.03 (0.70-1.53)	0.870
**Current alcohol**				
No (ref)	99 (49.5)	101 (50.5)	1	
Yes	110 (54.5)	92 (45.5)	1.22 (0.82-1.81)	0.320
**Sleep duration**				
3-4 hours	3 (60.0)	2 (40.0)	5.19 (0.80-33.80)	0.432
5-6 hours	26 (70.3)	11 (29.7)	**3.29 (1.34-8.09)**	0.131
7-8 hours	113 (47.7)	124 (52.3)	1.13 (0.57-2.22)	0.222
9 or more hours (ref)	67 (63.9)	56 (45.5)	1	
**Perceived enough sleep**				
Yes (ref)	161 (46.3)	187 (53.7)	1	
No	48 (88.9)	6 (11.1)	**9.29 (3.88-22.28)**	<0001
**Sleep quality**				
Good (ref)	135 (43.8)	173 (56.2)	1	
Intermediate	39 (69.6)	17 (30.4)	**2.94 (1.59-5.42)**	0.001
Poor	35 (92.1)	3 (7.9)	**14.9 (4.50-49.66)**	<0.001
**Occupational factors**				
**Type of construction work**				
Manual labor (ref)	96 (57.8)	70 (42.2)	1	
Bricklayer	56 (53.8)	48 (46.2)	0.85 (0.52-1.39)	0.521
Internal finish worker	29 (49.2)	30 (50.8)	0.71 (0.39-1.28)	0.250
Armature fixing	25 (51.0)	24 (49.0)	0.76 (0.40-1.44)	0.399
Other	3 (12.5)	21 (87.5)	**0.10 (0.03-0.36)**	<0.001
**Working hours per day**				
8 hours or less (ref)	88 (56.1)	69 (43.9)	1	
9 to 10 hours	50 (45.5)	60 (54.5)	0.65 (0.40-1.07)	0.089
11 and above	71 (52.6)	64 (47.4)	0.87 (0.55-1.38)	0.554
**Work experience**				
5 years or less (ref)	84 (41.6)	118 (58.4)	1	
More than 5 years	125 (62.5)	75 (37.5)	**2.34 (1.57-3.49)**	<0.001
**Psychosocial factors**				
**Depression**				
Absent (ref)	170 (51.1)	163 (48.9)	1	
Present	39 (56.5)	30 (43.5)	1.25 (0.74-2.10)	0.408
**Anxiety**				
Absent (ref)	161 (49.5)	164 (50.5)	1	

Present	48 (62.3)	29 (37.7)	**1.69 (1.01-2.81)**	0.043
**Stress**				
Absent (ref)	172 (51.6)	164 (48.8)	1	
Present	37 (56.1)	29 (43.9)	1.22 (0.72-2.07)	0.469
**Satisfaction**				
High (ref)	153 (49.7)	155 (50.3)	1	
Low	56 (59.6)	38 (40.4)	1.49 (0.93-2.39)	0.093
**Work-family life balance**				
High	133 (50.0)	133 (50.0)	1	
Low	76 (55.9)	60 (44.1)	1.27 (0.84-1.92)	0.264
**Job insecurity**				
Low (ref)	104 (47.3)	116 (52.7)	1	
High	105 (57.7)	77 (42.3)	**1.52 (1.02-2.26)**	0.037
**Hostile work environment**				
No (ref)	178 (50.7)	173 (49.3)	1	
Yes	31 (60.8)	20 (39.2)	1.51 (0.83-2.74)	0.23

***N:** Total frequency; **n**: frequency; **%**: percentage; **CI**: Confidence interval; **ref**: reference group*

***Bold** crude odds ratio indicates significant at 5% level of significance.*

Multivariate logistic regression in Table 4 revealed that the odds of having LBP was higher among females (AOR=2.42; 95%CI: 1.12-5.23), those living below poverty-line (AOR=2.35; 95%CI: 1.32-4.19), those with more than five years of work experience (AOR=1.66; 95%CI: 1.01-2.73) and those with intermediate sleep quality (AOR=2.06; CI: 1.03-4.11).

Table 4. Multivariate logistic regression analysis to determine independent predictor of LBP (N=402)

Variables	Category	Adjusted Odds Ratio (95% CI)	p-value
**Gender**	Male (ref)	1	
	Female	**2.42 (1.12-5.23)**	**0.024**
**Age**	18-30 (ref)	1	
	30-40	1.32 (0.78-2.27)	0.299
	Above 40	1.52 (0.76-3.06)	0.241
**Poverty**	Above poverty line(ref)	1	
	Below poverty line	**2.35 (1.32-4.19)**	**0.004**
**Comorbidity**	Absence(ref)	1	
	Presence	**2.18 (1.02-4.65)**	**0.045**
**Perceived enough sleep**	Yes(ref)	1	
	No	**4.93 (1.56-15.60)**	**0.007**
**Sleep quality**	Good(ref)	1	
	Intermediate	**2.06 (1.03-4.11)**	**0.041**
	Poor	3.89 (0.91-16.69)	0.067
**Type of construction worker**	Manual labor(ref)	1	
	Bricklayer	1.02 (0.54-1.93)	0.955
	Internal finish worker	0.78 (0.37-1.65)	0.518
	Armature fixing worker	1.63 (0.76-3.47)	0.208
	Other(painter/electrician)	**0.188 (0.05-0.72)**	**0.014**
**Work experience**	5 years or less(ref)	1	
	More than 5 years	**1.66 (1.01-2.73)**	**0.045**
**Anxiety**	Absence(ref)	1	
	Presence	1.33 (0.73-2.42)	0.355
**Job insecurity**	Low(ref)	1	
	High	0.84 (0.49-1.44)	0.522
**Job satisfaction**	High(ref)	1	
	Low	0.83 (0.44-1.59)	0.578
**Constant**	-	0.401	0.007

***CI**: Confidence interval; **ref**: reference group*

***Bold** adjusted odds ratio indicates significant at 5% level of significance.*

Protective measures used by Construction workers

The majority of the participants without LBP (95.0%) in the last one year did not use any protective measures to prevent low back pain. Only 5.0% of participants used some protective measures of which 4.0% used patuka (special piece of cloth worn around the waist) and remaining 1.0% did exercise.

The majority of the participants with low back pain (61.2%) did nothing against low back pain in past one year. 28.7% used patuka, 8.6% took medication prescribed by doctors, 7.2% took medication from the pharmacy, 2.4% had physiotherapy and 1.4% did the exercise to manage low back pain.

Discussion

This study aimed to find out prevalence of LBP, factors associated with LBP among construction workers of Nepal and find out measures taken to prevent and manage LBP.

Prevalence of Low Back Pain

The overall one-year prevalence of LBP among construction workers was 52.0%. Our findings are consistent with the previous cross-sectional questionnaire based study that reported a similar one year prevalence of LBP among construction workers(13,28). In contrast, Reddy et al [31] and Kaneda et al [32] reported lower prevalence of LBP with 20.8% and 29.3% respectively among construction workers. Bodhare *et al*[33] *and* Araújo et al [34] reported the highest one-year prevalence of LBP with 92.0% and 71.4% respectively among construction workers compared to present study.

The one-year prevalence of low back pain among male construction workers in the current study was 48.1%. Our finding are similar with cross-sectional studies conducted by Alghadir [14] et al. and Ueno et al. [11] among male construction workers which reported 50.0% and 53.2% respectively. Telaprolu *et al* [16] reported one-year prevalence of LBP to be 44.1% among women construction workers which was much lower compared the present study.

The prevalence of low back pain in the general population of low-income countries ranged from 0 to 16%[35] which was far lower than the findings of the present study (52.0%) among the construction workers. A study in Nepal found 36.2% one-year prevalence of LBP among farmers [36] which was less than the result of the present study. Though both agriculture and

construction works involved hard physical labor and mechanical nature, the LBP is more common among construction workers than farmers.

Prevalence of chronic low back pain

The prevalence of CLBP reported in this study is consistent with the findings of Latza *et al* which reported the one-year prevalence of CLBP to be 15.4% among construction workers[12]. This study reported the prevalence of CLBP two times the prevalence stated by Holmstrom *et al* [15].

Risk factors for low back pain

Socio-demographic risk factors

One-year prevalence of LBP and CLBP were higher among females compared to male construction workers (LBP: 72.0%vs 48.0%, CLBP: 26.2% vs 11.9%). The findings showed an agreement with several studies conducted among construction workers and the general population[11,15,32,33,37–39]. In contrast, there was no difference in the prevalence of LBP between males and females in a study carried among Japanese construction workers[32]. The difference in the prevalence of low back pain may be due to sex differences that could be related to gonadal steroid hormones like testosterone and estradiol modulate sensitivity to analgesia and pain[40].

The age of the participants with LBP was significantly higher than those without LBP. Increasing age was significant for the outcome of low back pain in the current study. The role of age on LBP was also reported by Holmström *et al*[15], Kaneda *et al*[32], and Ueno *et al*

[11]. This is because, as population ages, low back pain increases substantially due to the deterioration of the intervertebral discs in older people [8].

In the present study, the married have 3.76 times the odds of LBP and 5.18 times the odds of CLBP in comparison to unmarried. This finding was congruent with the finding made by Kaneda *et al* where unmarried participants were 0.70 times less likely to have LBP [32]. This relationship might be because married participants have a significantly higher age than unmarried participants.

In the current study, LBP was found to be significantly higher among construction workers living below poverty line. This association can be explained in two possible ways. One is the inability to perform productive work due to the presence of low back pain may be driving workers into poverty. Another is poor nutrition due to poverty might be leading to LBP.

Lifestyle risk factors

Alcohol: Several studies have not listed alcohol intake as a risk factor of LBP but a study have shown that continued alcohol intake was related to the deterioration of muscle strength and appearance of histological injury to muscle[41]. So alcohol consumption was taken as one of the associated factors for LBP but current study did not find any significant association of LBP with alcohol consumption like in the study by Ueno *et al* [11].

Smoking: Researchers don't take smoking as a cause of musculoskeletal pain but as a confounding factor[11] as smoking is associated with job dissatisfaction, job insecurity, anxiety, stress, and depression[42]. A population-based study showed that smokers were 1.23

times likely to develop low back pain than non-smokers[38]. In contrast, the present study did not find any association between smoking and LBP among construction workers. Further detailed studies are necessary to determine the causal relationship between LBP and smoking.

Obesity: Some studies showed that increased overweight is associated with lumbosacral reticular pain[43] and an increase in BMI is associated with lumbar disc herniation which is the important cause of LBP[44]. Deyo *et al* showed a significant increase in the prevalence of LBP with an increase in body mass index[45]. But this study, similar to Chung et al [46], failed to find any association between LBP and obesity among construction workers.

Sleep: Pain has been reported to have bi-directional relationship with sleep - pain hinders sleep and sleep disturbances reduce the pain threshold and mental capacity to manage pain [47]. This study failed to determine any association with LBP.

Occupational risk factors

In the present study, the prevalence of LBP and CLBP was significantly lower among painter and electrician in comparison to manual labors and other types of construction workers. This might be because of the difference in the nature of work and working posture among the construction workers. Manual labors, bricklayers and armature fixing workers are more prone to working postures like bending heavily with one’s trunk, bending and twisting simultaneously with one’s trunk, a bent and twisted posture for long periods, and making repetitive movements with the trunk or exposed to vibrations which are known occupational risk factors for low back pain[8].

Similar to the findings of Kaneda et al[32], work experience was significantly higher among workers with LBP compared to those without LBP in this study.

There have been various reports on the strong relationship between the duration of work and the prevalence of LBP[32,48,49]. In contrast to these studies, our study did not find any association with the duration of work.

Psychosocial risk factors

Depression, anxiety, and Stress: Psychosocial factors have been found to play an important role in the development of LBP[50]. Previous studies stated that psychological distress was associated with low back pain among various groups of working population[50–52] including construction workers[53]. Depression and anxiety are considered as an internalizing type of psychological distress [54]. Some researchers suggested the association of psychological distress and low back pain might be due to the influence of work-related psychosocial factors [50–53]. In the present study, there was a significant association of chronic low back pain with psychological distress, depression and anxiety; and low back pain with anxiety. In agreement with our findings, a study by Frymoyer *et al and* Abolfazl *et al* also found an association between depression and anxiety with low back pain[55,56].

Job Insecurity: Some studies have indicated musculoskeletal disorder including LBP as the damaging effect of job insecurity[29]. It is believed that mental strain linked with job insecurity may indirectly lead to "physiological vulnerability" which, in turn, may contribute to low back pain[26]. Hence job insecurity was taken as independent variable in the present study. We

found significant association between job insecurity and LBP among construction workers in univariate analysis but there existed no significant association after adjusting other variables.

Work-family life imbalance: Work-family imbalance is postulated to cause mental strain which in turn results in muscle tension or other physiological processes that might exacerbate low back pain[57]. In addition, work-family life imbalance is believed to drain psychological and physical resources leading to unhealthy behaviors, including alcohol and tobacco use and decreased leisure-time physical activity which is expected to cause LBP [58]. But this study could not find significant association of work-family balance with low back pain among construction workers.

Hostile Work Environment and Job Satisfaction: Researcher have hypothesized that job dissatisfaction and exposure to hostile work environment leads to mental strain which in turn alters biochemical and physiological processes of pain perception leading to Musculoskeletal pain including LBP [29,57]. But our study could not find independent association of exposure to hostile work environment and job dissatisfaction with LBP among construction workers.

Protective measures

A study through observation of intra-abdominal pressure and the lumbosacral compression force confirmed that on wearing patuka or lumbar supporter might be accountable for the low incidence of LBP [59]. But very few construction workers, 28.7% among those with LBP and 4.0% among those without LBP were using patuka to prevent or manage low back pain. Similar

to the present study, construction workers of Japan also poorly use lumbar supporter as protective equipment for LBP[32]. According to a study by Shrestha *et al,* safety practices of Nepalese construction projects, mainly the use of personal protective equipment, is gradually growing[60]. Though the use of PPE is growing in the context of Nepal, construction workers in the present study were poorly using PPE like patuka or belt to prevent LBP.

Most of the construction workers in our study felt LBP as a consequence of hard work and subsides immediately after taking rest which results in the reduction of healthcare utilization among construction workers for LBP. From this findings, we can support the statement that low back pain is a neglected problem among construction workers in the context of Nepal.

Limitations

Though this study did best to find the prevalence of low back pain and factors associated with low back pain among construction workers, it is not free from limitations. Because of the cross-sectional nature of the study, the directionality of the risk associations cannot be established. Several factors like awkward, static and dynamic working posture; rare factors like osteoporosis, prolonged corticosteroid use, vertebral infections, and tumors were not collected in the present study. The assessment of the psychosocial risk factors like job insecurity, job satisfaction, work-family life balance and exposure to a hostile work environment was done in this study using single items for each psychosocial domain.

Conclusion

It can be concluded that the prevalence of low back pain in the past year was high among construction workers. Factors like gender, poverty, co-morbidity, sleep quality, and work experience were found to be independently associated with the presence of LBP in the past year. Sleep quality and anxiety were independently associated with chronic low back pain. Health seeking behaviour for low back pain was very poor among construction workers. A high proportion of construction workers did nothing to prevent or manage low back pain. The findings of the present study are applicable to develop public health and occupational health strategies, programs and guidelines in Nepal to counteract the problem of LBP among construction workers and save the potential of labor workforce.

Acknowledgments

We would like to acknowledge all the people who directly or indirectly contributed to the present study and all the study participants who shared their valuable time with us.

Conflict Of Interest

There are no conflict of interests

References

1. National Planning Commission. Report on the Nepal Labour Force Survey 2017/18. Central Bureau of Statistics; 2019. Available: hhss@cbs.gov.np

2. Louw QA, Morris LD, Grimmer-Somers K. The prevalence of low back pain in Africa: a systematic review. BMC Musculoskelet Disord. 2007;8: 1–14.

3. Sharma S, Traeger AC, Mishra SR, Sharma S, Maher CG. Delivering the right care to people with low back pain in low- and middle-income countries: the case of Nepal. J Glob Health. 2019;9: 010304. doi:10.7189/jogh.09.010304

4. Hoy D, Brooks P, Blyth F, Buchbinder R. The epidemiology of low back pain. Best Pract Res Clin Rheumatol. 2010;24: 769–781.

5. Lambeek LC, Bosmans JE, Van Royen BJ, Van Tulder MW, Van Mechelen W, Anema JR. Effect of integrated care for sick listed patients with chronic low back pain: economic evaluation alongside a randomised controlled trial. Bmj. 2010;341: c6414.

6. Thelin A, Holmberg S, Thelin N. Functioning in neck and low back pain from a 12-year perspective: a prospective population-based study. J Rehabil Med. 2008;40: 555–561.

7. Kent PM, Keating JL. The epidemiology of low back pain in primary care. Chiropr Osteopat. 2005;13: 1–7.

8. Duthey B. Background Paper 6.24 Lower Back Pain. 2013.

9. Vrbanić TS-L. Križobolja-od definicije do dijagnoze low back pain-from definition to diagnosis. Reumatizam. 2011;58.

10. Yoshida T, Goto M, Nagira T, Ono A, Fujita I, Goda S, et al. Studies on low back pain among workers in small scale construction companies. Jpn J Ind Health. 1971;13: 37–45.

11. Ueno S, Hisanaga N, Jonai H, Shibata E, Kamijima M. Association between musculoskeletal pain in Japanese construction workers and job, age, alcohol consumption, and smoking. Ind Health. 1999;37: 449–456.

12. Latza U, Pfahlberg A, Gefeller O. Impact of repetitive manual materials handling and psychosocial work factors on the future prevalence of chronic low-back pain among construction workers. Scand J Work Environ Health. 2002; 314–323.

13. Biswas G, Ali M, Bhattacharya R. Occupational health risk of construction workers: A sample based study. Int J Pharma Res Bio-Sci. 2016;5: 129–41.

14. Alghadir A, Anwer S. Prevalence of musculoskeletal pain in construction workers in Saudi Arabia. Sci World J. 2015;2015.

15. Holmström EB, Lindell J, Moritz U. Low Back and Neck/Shoulder Pain in Construction Workers: Occupational Workload and Psychosocial Risk Factors. Spine. 1992;17: 663–671. doi:10.1097/00007632-199206000-00005

16. Telaprolu N, Lal B, Chekuri S. Work related musculoskeletal disorders among unskilled Indian women construction workers. Age Yrs. 2013;22: 36–4.

17. Gallagher S. Reducing low back pain and disability in mining. 2008.

18. Deacon C, Smallwood J, Haupt T. The health and well-being of older construction workers. Elsevier; 2005. pp. 172–177.

19. Himalowa S. The Effect of Occupational -related low back pain on functional activities among male construction workers. Thesis, University of the Western Cape. 2010.

20. Reese CD, Eidson JV. Handbook of OSHA construction safety and health. Crc Press; 2006.

21. Training of Trainers on Work Improvement in Small Construction Sites (WISCON). 22 Mar 2017 [cited 21 Jun 2020]. Available: http://www.ilo.org/kathmandu/whatwedo/eventsandmeetings/WCMS_548386/lang--en/index.htm

22. Biswas G, Bhattacharya A, Bhattacharya R. Occupational health status of construction workers: A review. Int J Med Sci Public Health. 2017;6: 669–675.

23. World Bank. World Bank in Nepal-Overview. In: World Bank [Internet]. [cited 6 Jun 2020]. Available: https://www.worldbank.org/en/country/nepal/overview

24. Pan W-H, Yeh W-T. How to define obesity? Evidence-based multiple action points for public awareness, screening, and treatment: an extension of Asian-Pacific recommendations. Asia Pac J Clin Nutr. 2008;17: 370.

25. Karki K, Dahal B, Regmi A, Poudel A, Gurung Y. WHO STEPS Surveillance: Non Communicable Diseases Risk Factors Survey. 2008. Kathmandu Minist Health Popul GoN Soc Local Integr Dev Nepal SOLID Nepal WHO. 2008.

26. Yang H, Haldeman S. Behavior-related factors associated with low back pain in the US adult population. Spine. 2018;43: 28–34.

27. Lovibond SH, Lovibond PF. Manual for the depression anxiety stress scales. Psychology Foundation of Australia; 1996.

28. Tonsing KN. Psychometric properties and validation of Nepali version of the Depression Anxiety Stress Scales (DASS-21). Asian J Psychiatry. 2014;8: 63–66.

29. Yang H, Haldeman S, Lu M-L, Baker D. Low back pain prevalence and related workplace psychosocial risk factors: a study using data from the 2010 National Health Interview Survey. J Manipulative Physiol Ther. 2016;39: 459–472.

30. Godwin HC, Ndubueze DU. A Survey on the prevalence of musculoskeletal disorders among building construction workers in Anambra State. Int J Adv Engg TechIVIVOct-Dec. 2013;11: 15.

31. Reddy GM, Nisha B, Prabhushankar TG, Vishwambhar V. Musculoskeletal morbidity among construction workers: A cross-sectional community-based study. Indian J Occup Environ Med. 2016;20: 144.

32. Kaneda K, Shirai Y, Miyamoto M. An epidemiological study on occupational low back pain among people who work in construction. J Nippon Med Sch. 2001;68: 310–317.

33. Bodhare T, Valsangkar S, Bele S. An epidemiological study of work-related musculoskeletal disorders among construction workers in Karimnagar, Andhra Pradesh. Indian J Community Med Off Publ Indian Assoc Prev Soc Med. 2011;36: 304.

34. Araújo SP, Carvalho LN, Martins ÉS. Lower back pain and level of disability amongst construction workers. Fisioter Em Mov. 2016;29: 751–756.

35. Volinn E. The epidemiology of low back pain in the rest of the world: a review of surveys in low-and middle-income countries. Spine. 1997;22: 1747–1754.

36. Mahto PK, Gautam BB. Prevalence of work-related musculoskeletal disorders in agricultural farmers of Bhaktapur District, Nepal. Int J Occup Saf Health. 2018;8: 3–7.

37. Shrestha B, Niraula S, Khanal G, Karn N, Chaudhary P, Rijal R, et al. Epidemiology of back pain in the teaching districts of BP Koirala Institute of Health Sciences. Health Renaiss. 2011;9: 152–156.

38. Dijken C, Fjellman-Wiklund A, Hildingsson C. Low back pain, lifestyle factors and physical activity: A population based-study. J Rehabil Med. 2008;40: 864–869. doi:10.2340/16501977-0273

39. Ekpenyong CE, Inyang UC. Associations Between Worker Characteristics, Workplace Factors, and Work-Related Musculoskeletal Disorders: A Cross-Sectional Study of Male Construction Workers in Nigeria. Int J Occup Saf Ergon. 2014;20: 447–462. doi:10.1080/10803548.2014.11077057

40. Craft RM, Mogil JS, Aloisi AM. Sex differences in pain and analgesia: the role of gonadal hormones. Eur J Pain. 2004;8: 397–411. doi:10.1016/j.ejpain.2004.01.003

41. Estruch R, Sacanella E, Fernandez-Sola J, Nicolas JM, Rubin E, Urbano-Marquez A. Natural History of Alcoholic Myopathy: A 5-Year Study. Alcohol Clin Exp Res. 1998;22: 2023–2028. doi:10.1111/j.1530-0277.1998.tb05911.x

42. Dempsey PG, Burdorf A, Webster BS. The Influence of Personal Variables on Work-Related Low-Back Disorders and Implications for Future Research: J Occup Environ Med. 1997;39: 748–759. doi:10.1097/00043764-199708000-00010

43. Shiri R, Karppinen J, Leino-Arjas P, Solovieva S, Varonen H, Kalso E, et al. Cardiovascular and lifestyle risk factors in lumbar radicular pain or clinically defined sciatica: a systematic review. Eur Spine J. 2007;16: 2043–2054. doi:10.1007/s00586-007-0362-6

44. Schumann B, Bolm-Audorff U, Bergmann A, Ellegast R, Elsner G, Grifka J, et al. Lifestyle factors and lumbar disc disease: results of a German multi-center case-control study (EPILIFT). Arthritis Res Ther. 2010;12: R193. doi:10.1186/ar3164

45. Deyo RA, Bass JE. Lifestyle and Low-Back Pain: The Influence of Smoking and Obesity. Spine. 1989;14: 501–506. doi:10.1097/00007632-198905000-00005

46. Chung JWY, So HCF, Yan VCM, Kwok PST, Wong BYM, Yang JY, et al. A Survey of Work-Related Pain Prevalence Among Construction Workers in Hong Kong: A Case-Control Study. Int J Environ Res Public Health. 2019;16: 1404. doi:10.3390/ijerph16081404

47. Schuh-Hofer S, Wodarski R, Pfau DB, Caspani O, Magerl W, Kennedy JD, et al. One night of total sleep deprivation promotes a state of generalized hyperalgesia: A surrogate

pain model to study the relationship of insomnia and pain: Pain. 2013;154: 1613–1621. doi:10.1016/j.pain.2013.04.046

48. Ohara H. Epidemiolog of Occupational low Back Pain. JJT O M. 1993;42: 413–419.

49. Svensson HO, Andersson GB. Low-Back Pain in 40− to 47-Year-Old Men: Work History and Work Environment Factors. Spine. 1983;8: 272–276.

50. Linton SJ. A review of psychological risk factors in back and neck pain. Spine. 2000;25: 1148–1156.

51. Feyer A-M, Herbison P, Williamson AM, de Silva I, Mandryk J, Hendrie L, et al. The role of physical and psychological factors in occupational low back pain: a prospective cohort study. Occup Environ Med. 2000;57: 116–120.

52. Leino P, Magni G. Depressive and distress symptoms as predictors of low back pain, neck-shoulder pain, and other musculoskeletal morbidity: a 10-year follow-up of metal industry employees. Pain. 1993;53: 89–94.

53. Jacobsen HB, Caban-Martinez A, Onyebeke LC, Sorensen G, Dennerlein JT, Reme SE. Construction workers struggle with a high prevalence of mental distress and this is associated with their pain and injuries. J Occup Environ Med Coll Occup Environ Med. 2013;55: 1197.

54. Moore SA, Dowdy E, Furlong MJ. Using the Depression, Anxiety, Stress Scales–21 with US adolescents: An alternate models analysis. J Psychoeduc Assess. 2017;35: 581–598.

55. Frymoyer J, Pope M, Clements JH, Wilder DG, MacPherson B, Ashikaga T. Risk factors in low-back pain. An epidemiological survey. JBJS. 1983;65: 213–218.

56. Rahimi A, Vazini H, Alhani F, Anoosheh M. Relationship between low back pain with quality of life, depression, anxiety and stress among emergency medical technicians. Trauma Mon. 2015;20.

57. Sauter SL, Swanson NG. An ecological model of musculoskeletal disorders in office work. Biomech Psychosoc Asp Musculoskelet Disord Off Work. 1996; 3–21.

58. Hammer LB, Sauter S. Total worker health and work–life stress. J Occup Environ Med. 2013;55: S25–S29.

59. Shah R. The Nepalese patuka in the prevention of back pain. Int Orthop. 1994;18: 288–290.

60. Shrestha S, Shrestha HM. Construction Safety Measures Implementation Status in Nepal. J Adv Civ Eng Manag. 2019;2.
