# Supplementary material for: Factors associated with low back pain among construction workers in Nepal: A cross-sectional study
Source: PLoS One. 2021 Jun 1;16(6):e0252564. doi: 10.1371/journal.pone.0252564 (PMC8168885; doi:10.1371/journal.pone.0252564)
Supplement: Attachment — Submitted filename: Response to Reviewers.docx [file pone.0252564.s002.docx]

**Response to Reviewers**

**08/04/2021**

**To**

**Editors and team**

**PLOS ONE journal**

**Subject: Re-submission of an original research article to PLOS ONE journal addressing the reviewers comment**

Dear sir/madam,

It is our pleasure to re-submit to you and the editorial teams our manuscript entitled **“Factors associated with low back pain among construction workers in Nepal: A cross-sectional study”**. This study aimed to determine the prevalence of Low back pain, one of the neglected non communicable diseases and factors associated with it among construction workers.

The answers to the reviewer’s (Reviewer 2) comments are addressed individually below:

- Reference pattern in 49-56 lines are (?). It is abruptly starting from 21? And it starts directly from sentence line 64?
- **The references are updated as per the guideline of the journal**
- Line 103- why DASS was used? Nothing mentioned in objectives about it.
- **DASS is now mentioned in the objectives too**
- Line 113- why mentioned abruptly about CLBP. Nowhere has been ever talked about these types. Never mentioned in introduction clearly except abruptly mentioned once.
- **CLBP is a Chronic Low back pain. We’ve assessed CLBP among construction workers but with discussion with co-authors we decided to remove CLBP from the present manuscript as it is confusing. We will submit supplementary article of this manuscript if accepted.**
- Line 140- define others (you have those answers for others… mentioned here )
  - **Other may include Glaziar, dry wall installer, and stone wall builder. Other is removed from the sentence as only construction workers mentioned in the list are enrolled in the present study.**
- Line 198- what do you mean by age limitation? As you have had exclusion criteria for age
  - **Yes, <18 years age is the exclusion criteria. For the purpose of documentation, we included the percentage of ineligibility along with the reasons.**

**We’ve assessed total 456 workers of which 5.7% were of age less than 18 years working in construction worker and 3.3% of the participants could not be communicated due to language barrier (speaking Bhojpuri or Indian language).**

- Results: Line 197- why did you assess 456 when they did not meet the criteria? Explain sample size calculation

**I’ve added sample size calculation section. We visited all municipalities and checked for presence of construction sites and workers. And all who meet eligibility criteria were included in the study which resulted to count of 456 participant who were assessed.**

- Please explain the enrollment process of the study participants clearly.
  - **It is clarified**
- Who are others in Ethnicity? Correct it
  - **Gurung, Jirel, thami, chepang, Majhi etc were other ethnicity. It is now mentioned in the table.**
- What do you mean? (J-m)
  - Family Size 5 (4, 6)
  - Dependent family members 3 (2, 4)
  - Monthly income (participant) N.Rs.25000 (20000, 25250)
  - Monthly family income N.Rs. 45000 (30000, 55000)

**They are median and Interquartile range. It is now clarified in the table**

- Age at joining construction industry (year): it carries great value so it would be best if you could show table of age joining for below 18 years because 127 is a good number.
  - **The mean and standard deviation of age of joining construction industry presents in the manuscript.**
- Table 4: amazing that age is not the predictor of LBP .Maybe you should try regrouping the age. Generally age is the predictor.
  - **I tried to regroup and analyze previously but could not conclude age as predictor.**
- It was difficult to understand difference between LBP and CLBP in your study. Please elaborate.
  - **CLBP is removed from the study with the discussion between co-authors. We planned to submit another supplementary article related to CLBP**
- Line 401- Health seeking behavior for low back pain was very poor among construction workers. As nowhere in your study you have mentioned the pain score so you cannot make this statement. Pain scale would have been better to be used and explore grades of LBP. I think this study need this to make your study more valid.
  - **The statement is removed from the manuscript. Our study assessed the pain scale among participants with CLBP so Pain scale related information in not added in the manuscript.**
- You have so many factors included in tables. Please make it smart including only needy variables. Many variables are unnecessarily shown. You have too many unnecessary variables which are not needed for this study and have no direct connections. So for this article those variables are not needed.
  - **I’ve removed some less important variables from the tables.**
- Chronic back pain has good connection with Depression. Here mostly you have used LBP not CLBP. So you should use this cautiously when needed.
  - **CLBP is removed from the study with the discussion between co-authors. We planned to submit another supplementary article related to CLBP**
- No variables which show real relations with LBP such as “working posture”, times of bending, pushing, pulling, dragging, carrying and holding are not in this study. So as topic suggests “the factors associated” is not supported in real.
  - **They are included in the limitation of the study.**
- LBP is also strongly associated with the bed quality where workers sleep. No variables related to it show a bias too.
  - **Our study assess the sleep quality and perceived enough sleep in order to address the bed quality which is related to LBP.**

**Dr. Akina Shrestha**, Assistant Professor, Kathmandu University School of Medical Sciences and all co-authors reviewed and edited the manuscript. All changes made on the manuscript are track changed or highlighted. The track changed version and final version of the manuscript are submitted.

We look forward to your comments and remain with best regards;

**Yours sincerely,**

**Mr. Bikram Adhikari,**

MPH**,** School of Public Health and Community Medicine

B.P. Koirala Institute of Health Sciences
